# Genome-wide uniparental disomy screen in human discarded morphologically abnormal embryos

**DOI:** 10.1038/srep12302

**Published:** 2015-07-21

**Authors:** Jiawei Xu, Meixiang Zhang, Wenbin Niu, Guidong Yao, Bo Sun, Xiao Bao, Linlin Wang, Linqing Du, Yingpu Sun

**Affiliations:** 1 Center for Reproductive Medicine, The First Affiliated Hospital of Zhengzhou University, Zhengzhou, People’s Republic of China

## Abstract

Uniparental disomy (UPD) has been shown to be rare in human normal blastocysts, but its frequency in discarded morphologically abnormal embryos and its relevance to embryonic self-correction of aneuploid remains unknown. The aim of this study was to detect UPD in discarded morphologically abnormal embryos. Both discarded morphologically abnormal embryos, including zero-pronuclear zygotes (0PN), one-pronuclear zygotes (1PN), three-pronuclear zygotes (3PN) and 2PN embryos scored as low development potential were cultured into blastocysts then underwent trophectoderm biopsy. Genome-wide UPD screening of the trophectoderm of 241 discarded morphologically abnormal embryo sourced blastocysts showed that UPD occurred in nine embryos. Five embryos exhibited UPDs with euploid chromosomes, and four displayed UPDs with chromosomal aneuploid. The percentage of UPDs among the morphologically abnormal sourced blastocysts was 3.73%, which is significant higher than the percentage observed in normal blastocysts. The frequency of UPD in 3PN-sourced blastocysts was 7.69%, which is significantly higher than that in normal blastocysts. This study provides the first systematic genome-wide profile of UPD in discarded morphologically abnormal embryos. Our results indicated that UPD may be a common phenomenon in discarded morphologically abnormal embryos and may be relevant to human embryonic self-correction.

Uniparental disomy (UPD) occurs when two identical (isodisomy) or homologous (heterodisomy) chromosomes are inherited from one parent^1^. It occurs as a random event during the formation of oocytes or sperm, early foetal development, or trisomic rescue^2^,^3^. In heterodisomic UPD, two distinct homologous chromosomes are received from one parent, indicating that an error took place during meiosis I. Isodisomic UPD occurs when two identically replicated copies are inherited from a single homologue of a specific chromosome, which implies that an error took place during either meiosis II or postzygotic chromosomal duplication. The prevalence of UPD in newborns has been estimated to be approximately 0.03%^3^,^4^.

“Embryo self-correction” of aneuploid could hypothetically occur during development when an aneuploidy embryo shows a potential capacity for correcting to euploid by itself. Previous study showed that cleavage-stage aneuploidy embryos became euploid upon developing into blastocysts, which is considered to be the result of self-correction^5^. The self-correction of embryos with aneuploidy is thought to be a possible mechanism leading to the occurrence of UPD^5^,^6^,^7^,^8^, which usually occurred during *in vitro* fertilization. Monosomic duplication results in isodisomy, extruding an extra chromosome in the case of trisomy-correction leads to heterodisomy. SNP arrays can be used to evaluate embryonic self-correction mechanisms by detecting isodisomy and heterodisomy^9^. Previous study reported that UPD was extremely rare (approximately 0.06%) in human blastocysts, and thus, UPD screening before embryo implantation was considered unnecessary^10^. However, it is not clear whether UPD is rare or common in blastocysts that develop from discarded morphologically abnormal embryos, such as blastocysts developed from 3PN embryos^10^. UPD is thought to represent a possible outcome of putative embryo self-correction, which occurs in approximately 10%–71% of embryos according to data from *in vitro* fertilization. The aim of self-correction is to correct chromosomal abnormalities caused by mitotic nondisjunction, chromosome loss and chromosome gain. In particular, monosomic chromosome duplication and trisomic chromosome extrusion could explain the mechanism of UPD occurs^5^,^6^,^8^,^11^,^12^. Nevertheless, a mosaic exists in developing embryos^13^, and the proportion of UPD may therefore reflect the scale of the formerly chromosome mosaic. Based on this theory, significant chromosome abnormalities may cause embryonic developmental arrest even after self-correction. We concluded that UPD could be an outcome of abnormal chromosome embryo self-correction and would be detected in discarded morphologically abnormal embryos.

13 embryos harbouring 15 UPDs were discovered in 3,655 embryos from couples who underwent pre-implantation genetic screening (PGS), and the frequency ranged from 0 to 0.57% per chromosome^10^,^14^,^15^,^16^. However, UPDs in blastocysts are still rare compared with other chromosomal abnormalities, and it remains unknown how UPDs occur in embryos. Therefore, to determine whether UPD is a common phenomenon in discarded morphologically abnormal embryos, we performed SNP microarray-based 24 chromosome aneuploidy screening in embryos to determine the overall frequency of UPD in a large number of blastocysts developed from discarded morphologically abnormal embryos, which may support the hypothesis that UPD is a possible outcome of putative embryo self-correction.

## Results

### Characterization of the clinical patients in our study

Among the 241 trophectoderm-biopsied embryos from 169 couples evaluated in this study, the mean paternal age of the discarded embryos was 31 years (range, 23 to 42 years), and the mean maternal age of the UPD-containing embryos was 30 years (range, 23 to 42 years). The oocyte numbers and fertilization rates of the couples with UPD-containing embryos are provided in Table 1. UPD detection in our study was achieved without artificial selection for embryos collection.

### Validation of UPD detection using SNP microarray

**Figure 1:**
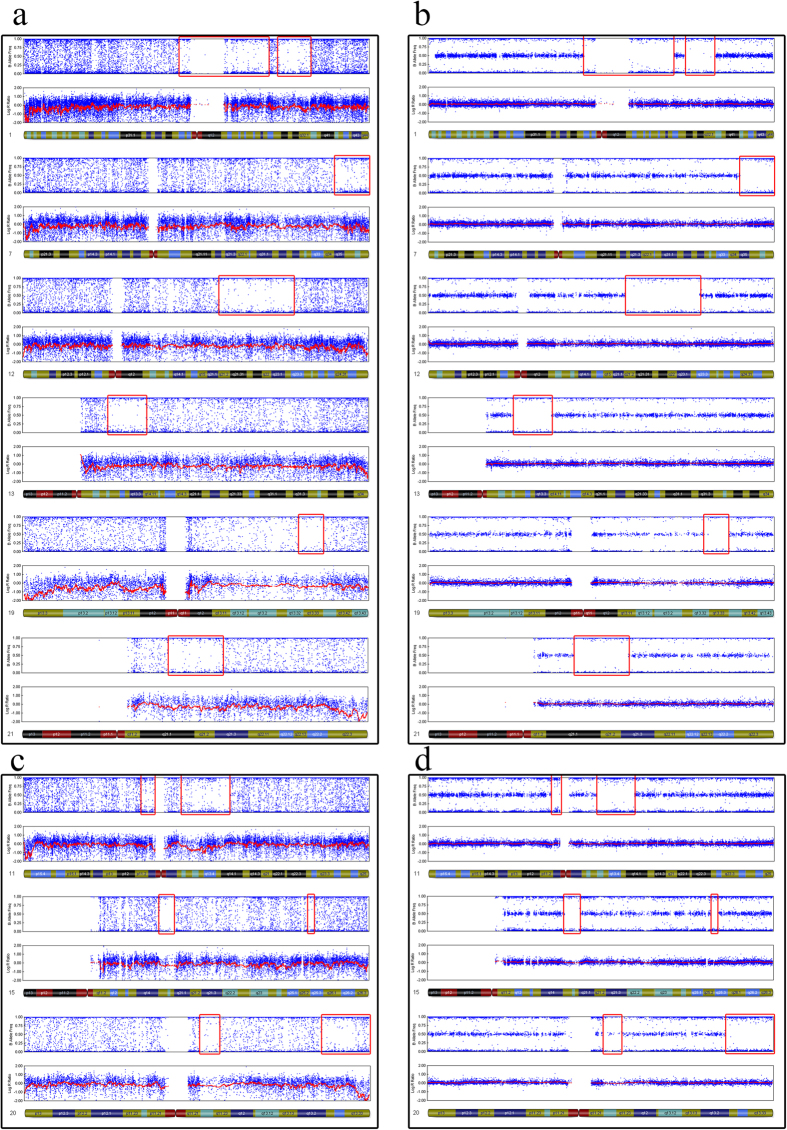
Comparison of SNP results obtained following the amplification of whole blood DNA and limited cell samples. Multi-cell and limited-cell results indicate the same UPD pattern on these chromosomes. **a**. UPDs detection on chromosome 1, 7, 12, 13, 19 and 21 using MDA-amplification production of limited cell. **b**. UPDs detection on chromosome 1, 7, 12, 13, 19 and 21 using global genomic DNA. **c**. UPDs detection of single cell on chromosome 11, 15 and 20. **d**. UPDs detection of multi cells on chromosome 11, 15 and 20.

**Figure 2:**
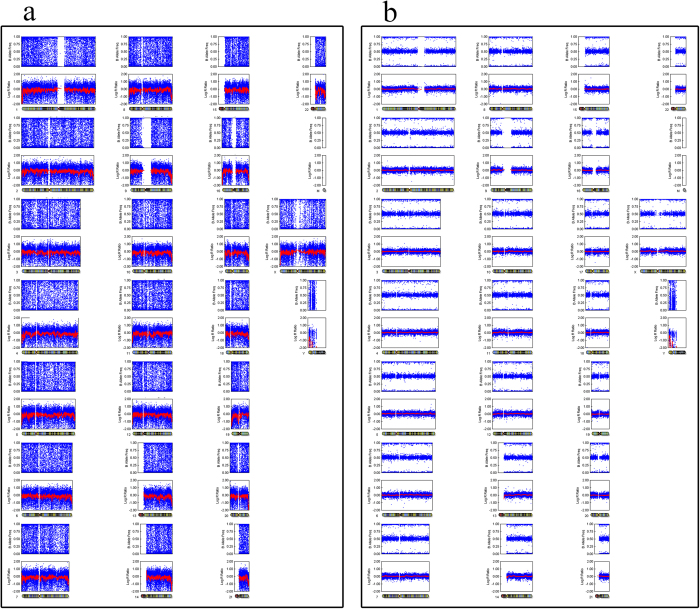
Normal female SNP array with two X chromosomes. Single-cell and multi-cell methods indicate the same karyotype. **a**. SNP array of limited cell MDA-amplification **b**. Genomic DNA SNP array result.

To validate the method (using MDA-amplified DNA and SNP array to detect UPD) used in our study, we selected 4 control blood samples and each with 5 replicates for MDA-amplification process. Two normal karyotypes, Arr (1–22) × 2,(XY) × 1, and Arr (1–22) × 2,(X) × 2, were used as negative controls, and two patients with UPD genotypes, [Arr (1–22) × 2, (XY) × 1,UPD(11)(q13.1-q14.1), UPD(15)(q15.1–q21.1),UPD(20)(q13.13-13.33)] and [Arr (1–22) × 2,(X) × 2 UPD(1)(p13.2–p25.1)(q25.3–q32.1),UPD(7)(q35-qter),UPD(12)(q21.2–q23.3),UPD(13)(q12.2–q14.1),UPD(19)(q13.3),UPD(21)(q21.1–q21.3)], were used as positive controls. The G banding karyotypes of these four patients were 46, XX and 46, XY. Genomic DNA was extracted from whole blood samples from each of the four patients for SNP microarray. Simultaneously, to emulate the quantity of cells obtained through trophectoderm biopsy, approximately 3 to 5 peripheral blood lymphocytes were prepared for MDA-amplification. After cell lysis, we used the same genome amplification method as trophectoderm biopsy to perform whole-genome amplification. We performed five repetitions of the amplification procedure and SNP array scan. GenomeStudio software was used to generate SNP genotype calls as previously described for embryos. We acquired the same results using whole blood DNA and limited cell amplification (Figs 1 and 2), which showed that the UPD detection method applied in this study is precise.

**Figure 3:**
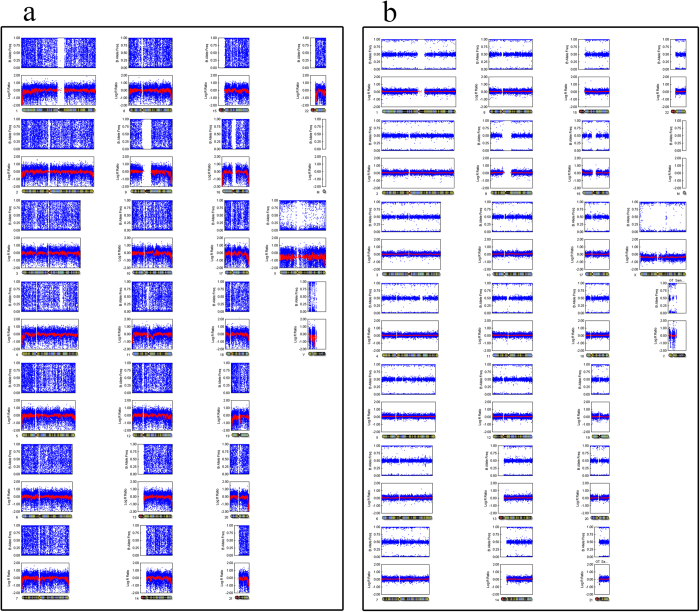
Normal male SNP array with one X chromosome and one Y chromosome. **a**. SNP array of limited cell MDA-amplification. **b**. Genomic DNA SNP array result.

In the validation group, control 1 harboured 7 UPDs located in 5 chromosomes. Control 2 exhibited 4 UPDs located in 3 chromosomes. These results are the same as those of the multi-cell of the same sample. The other two negative controls (one male and one female) showed no UPDs (Figs 1, 2 and 3). Both the samples using MDA-amplified DNA and those using global DNA from blood yielded the same results regarding UPD-containing regions.

### The frequency of genome-wide UPD in discarded morphologically abnormal embryos

**Figure 4:**
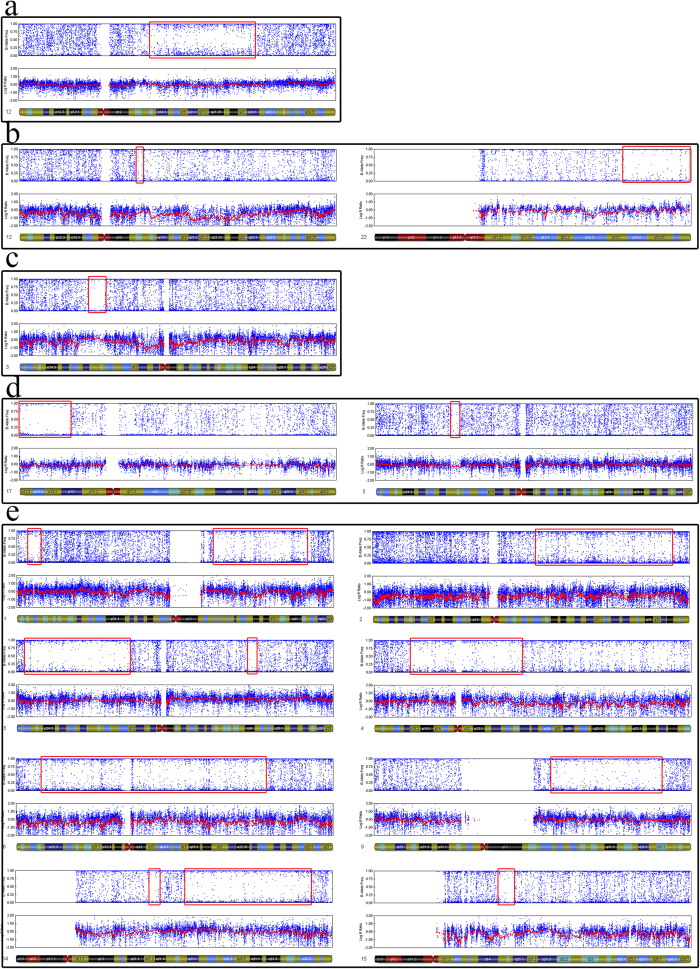
SNP array results for nine cases with UPDs. All of the results represent limited-cell data obtained following whole-chromosome amplification of embryo biopsy samples. Case 8 has UPDs on every autosome. **a**. case 1, **b**. case 2, **c**. case 3, **d**. case 4, **e**. case 5.

**Figure 5:**
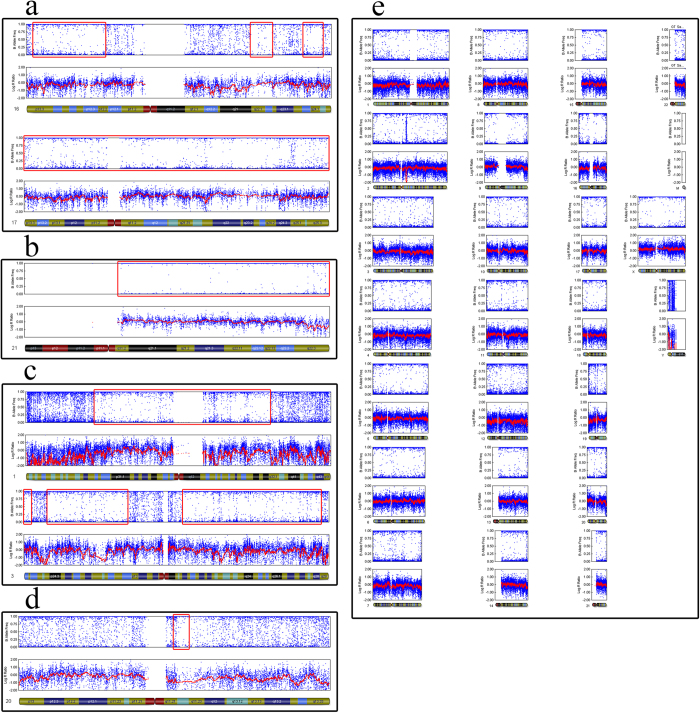
SNP array results for nine cases with UPDs. All of the results represent limited-cell data obtained following whole-chromosome amplification of embryo biopsy samples. **a**. case 6, **b**. case 7, **c**. case 8, **d**. case 9.

We identified 9 (3.73%) blastocysts contained isodisomy based on previously described criteria (Table 2). Among these embryos, 4 exhibited UPD without chromosome abnormalities. The other 5 embryos presented both UPD and other chromosome aneuploidy. In cases 2, 4B, 6 and 8, more than one chromosome was involved, with 8 chromosomes harbouring 14 UPDs in case 4B, showing a higher frequency genome-wide. Chromosomes 1 and 3 of case 6 exhibited 4 UPDs. In case 8, each chromosome, including the X chromosome, showed UPD. The other cases harboured only one UPD (Figs 4 and 5). The chromosome-specific frequency of UPD was 0.45% (49/11,004). The UPD identified in morphologically abnormal sourced blastocysts occurred most often on chromosomes 1, 3 and 17. The frequency of UPD in discarded morphologically abnormal embryos is significant higher than in normal embryos previously reported. In our study, we screened six 3PN sourced blastocysts, the results showed none were triploidy, indicating that genome-wide higher frequency UPDs may be an outcome of embryos self-correction.

### Imprinted genes located in UPD regions

To determine whether imprinted genes located in UPD regions are related to embryo development, we screened the genes located in the UPD regions using Karyostudio and the UCSC Genome Browser (Table 3). Then, we focused on three imprinted genes (*RBP5*, *BLCAP* and *NNAT*) located in UPD regions we detected in this study. UPD(12)(q13.13–q23.1) in embryo 1 covered the imprinted gene *RBP5*, which participates in the combination of retinol (vitamin A), influencing the enzymes controlling the conversion of the alcohol form of retinol: first to an aldehyde (retinaldehyde) and then to a carboxylic acid (retinoic acid; RA). During embryonic development, there is a concentration gradient of retinoic acid along the anterior-posterior (head-tail) axis. Retinoic acid influences the process of cell differentiation, and development of embryos^17^. UPD(20)(q11.22) located in embryo 7 covered the imprinted genes *BLCAP* and *NNAT. BLCAP* regulates cell proliferation and coordinates apoptosis and cell cycle progression via a novel mechanism that is independent of both p53/TP53 and NF-kappa-B^18^. *NNAT* participates in the maintenance of segment identity in the hindbrain and pituitary development, as well as the maturation and maintenance of the overall structure of the nervous system^19^. Based on the functions of these genes, we speculated that UPD might act as an independent factor to affect embryonic development via imprinted genes. Further studies need to explore the promoter DNA methylation of these imprinted genes located in UPD area.

## Discussion

The frequency of UPD in morphologically abnormal sourced blastocysts is unclear, whether UPD is relevant to embryo self-correction remains unknown. In the present study, we represented the largest sample of morphologically abnormal sourced blastocysts to evaluate the occurrence of UPD. We genome-wide screened UPD in 241 blastocysts developed from morphologically abnormal embryos using SNP arrays, and found that 9 of them contained UPDs, the rate is significantly higher than that of previously published data from normal embryos^14^. The rate of UPD in 3PN-sourced blastocysts was 7.69%, which is significantly higher than that in normal blastocysts. Meanwhile, the karyotype of it was not triploid supporting the hypothesis that UPD may be an outcome of embryo self-correction^5^,^6^,^8^,^11^,^12^.

Loss of heterozygosity (LOH) results in a loss of the gene and the surrounding area, and as one form of LOH, UPD can also cause gene deletions. UPD causes serious clinical symptoms via recessive mutations through the production of homozygosity, leading to abnormal genetic patterns^20^,^21^. Certain imprinted genes, such as *RBP5* and *BLCAP*, which were identified in the present study, participate in embryonic development, the abnormal status of these genes promoter DNA methylation may be a cause of embryo development arrest. LOH of imprinted genes will cause epigenetic changes, leading to abnormal expression^22^. However, these deletions are different from traditional genome copy number variants because the DNA copy number does not change. LOH is also observed in many types of cancer cells due to somatic recombination during mitosis. Furthermore, in chromosomally abnormal cell lines, LOH is frequently found in conjunction with mosaicism, which is also accompanied by phenotypic abnormalities^3^. As noted previously, some UPD-containing blastocysts harbour only a single UPD, while others also exhibit chromosome abnormalities such as trisomy^10^, terminal imbalances, deletions and duplications^14^; our data are consistent with these previously reported findings. Among the blastocysts evaluated in our study, 117 showed an abnormal karyotype, and 4 harboured UPDs (3.41%). Additionally, 5 of the 124 (4.03%) embryos with a normal karyotype among the morphologically abnormal discarded blastocysts exhibited UPD. This result illustrates that the occurrence of UPD may be independent of chromosomal abnormalities and indicates that UPD may be a result of embryo self-correction.

UPD can cause loss of parental/marital expression because UPD is inherited from only one parent and results in the corresponding human imprinting syndrome if the UPD is located on chromosome 7, 11 or 15^23^. Our data showed that the UPD identified in morphologically abnormal sourced blastocysts occurred most often on chromosomes 1, 3 and 17; the imprinted genes located in this UPD region are listed in Table 3. Further research is required to investigate the DNA methylation status of these genes during embryonic development. It is still unknown whether UPD is associated with intellectual disability in patients^24^, but in other imprinting disorders, such as Angelman syndrome (AS) and Prader-Willi syndrome (PWS), a high rate of UPD can be detected (7% for AS and 25% for PWS)^25^. However, we did not detect any imprinted genes in the UPD regions associated with AS and PWS in our study. A study that includes a larger sample size will be necessary to investigate the relationship between UPD and imprinted genes.

It can be concluded that UPD is a result of self-correction and genomic rearrangement^26^. The identification of one male day three embryo containing isodisomies on chromosomes 2, 15 and X was previously reported^14^. In contrast, we performed trophectoderm biopsy on day 5 or 6 for subsequent SNP array analysis. Compared with blastomere biopsy, trophectoderm biopsy collects more cells and provides a more informative result. Even using this method, we cannot avoid mosaicism between the inner cell mass and trophectoderm. However, all methods for trophectoderm karyotyping suffer from the possibility of karyotype mosaicism. Moreover, there is no evidence to indicate that chromosome variations among blastomeres do not occur in day three embryos. Additional research has focused on the differences between day three and day five biopsies and verified the phenomena of mosaicism and self-correction^8^,^11^. The investigation of UPD has contributed to our knowledge of embryonic development, especially regarding chromosomal mosaicism and self-correction.

## Methods

### Patient population

The study was approved by the Ethics Committee of The First Affiliated Hospital of Zhengzhou University, and written informed consent was obtained from all participants. The above methods were carried out in accordance with the approved guidelines. The study was undertaken with the understanding and appropriate informed consent of each participant.

Discarded embryos (abnormal fertilized embryos: 0PN, 1PN and 3PN; and normal fertilized embryos with abnormal morphology: 2PN) were used in our study. The embryos were obtained from couples who underwent fertility treatment between March 2014 and August 2014 at the Reproductive Medical Center of the First Affiliated Hospital of Zhengzhou University (Henan, China). Discarded embryos were collected from patients who were over 20 years old and had undergone routine ovarian hyperstimulation, routine oocyte retrieval and routine in-vitro fertilization (IVF) or intracytoplasmic sperm injection (ICSI). All included embryos were collected and cultured to the blastocyst stage under identical conditions. Trophectoderm biopsies were performed on the embryos as previously described, in addition to SNP microarray screening^27^. Embryos from oocyte donors were excluded; only fresh embryos were included. All subjects included in our study were informed consent.

### Isodisomy detection

Isodisomy was detected using a SNP array as previously described^15^. Limited cells from blastocysts were originally processed through whole-genome amplification using the MDA method (REPLI-g Midi Kit; QIAGEN 150045). The HumanCytoSNP-12v.21 array, which covers more than 220,000 markers, was employed in the present study to detect UPD in the genome amplification products from limited cells, and the raw data were analysed using GenomeStudio software (Illumina). All steps were carried out following the manufacturer’s protocols. Two independent parties analysed all of the data, and the results were presented under strict criteria.

### Validation of isodisomy detection

Our results were required to meet certain criteria to identify isodisomy, and negative and positive controls were included. DNA from both normal and UPD-containing whole blood samples was extracted using the QIAamp DNA Mini Kit (QIAGEN 51306), and the obtained DNA was then evaluated using an SNP array. Several peripheral blood lymphocyte cells from samples that had previously been diagnosed genetically, two that contained UPDs and two control samples that did not contain UPDs, were regarded as positive and negative controls and were successfully amplified using the same MDA method (REPLI-g Midi Kit; QIAGEN 150045). The samples were then screened using the SNP array^27^, which was replicated five times per sample. We compared the results obtained through MDA amplification of the limited cell samples with those of the whole blood DNA to validate the method applied in this study. This research was performed with institutional review board approval.

## Additional Information

**How to cite this article**: Xu, J. *et al*. Genome-wide uniparental disomy screen in human discarded morphologically abnormal embryos. *Sci. Rep*. **5**, 12302; doi: 10.1038/srep12302 (2015).

## Figures and Tables

**Table 1:** Basic characterization of the patients harbouring UPDs.

Case No.	Age		G band karyotype		Fertilization method	Oocyte number	Fertilization ratio
	Paternal	Maternal	Paternal	Maternal			
1	26	26	46,XY	46,XX	IVF	11	82%
2	32	30	46,XY	46,XX	IVF	9	89%
3	26	27	46,XY	46,XX	IVF	26	54%
4A	42	40	46,XY	46,XX	ICSI	5	40%
4B	42	40	46,XY	46,XX	ICSI	5	40%
5	23	23	46,XY	46,XX	IVF	12	75%
6	31	31	46,XY	46,XX	ICSI	10	20%
7	33	33	46,XY	46,XX	IVF	13	100%
8	30	24	46,XY	46,XX	ICSI	13	46%

**Table 2:** SNP microarray results of discarded morphologically abnormal embryos.

Case No.	Prokaryotic	SNP result	Sex chromosome
1	3PN	Arr UPD(12)(q13.13–q23.1)	XY
2	3PN	Arr −16,UPD(10)(q21.2–q22.2),UPD(22)(q13.1-qter)	XY
3	3PN	Arr UPD(3)(p21.33–p21.1)	XY
4A	3PN	Arr −1,−4,+8,−13,−15,−16,−19,−21,UPD(3)(p21.31-p21.1),UPD(17)(p12-pter)	XY
4B	2PN	Arr –Y,−2,−7,−8,−11,−13,−22,UPD(1)(p36.13-pter)(q21.3–q42.2),UPD(3)(p26.6-p13)(q24),UPD(4)(p15.31–q21.3),UPD(6)(pter-q23.3),UPD(9)(q21.2–q34.11),UPD(14)(q21.2–q21.3)(q22.3–q32.2),UPD(15)(q15–q15.3),UPD(16)(p12.2-pter)(q22.1-q22.2)(q23.3–q24.1),UPD(17)	XO
**5**	2PN	Arr −7,−12,−13,UPD(21)	XX
**6**	3PN	Arr UPD(1)(p32.2-p32.1),UPD(3)(pter-p26.2)(p24.3-p13)(q12.3-qter)	XX
7	2PN	Arr UPD(20)(q11.22)	XX
8	3PN	Arr UPD(1-22,X)	XX

**Table 3:** Imprinted genes located within the UPD regions detected in our study.

Case No.	SNP microarray karyotype	Imprinted gene
1	Arr UPD(12)(q13.13–q23.1)	*RBP5*
2	Arr −16,UPD(10)(q21.2–q22.2),UPD(22)(q13.1-qter)	*NA*
3	Arr UPD(3)(p21.33–p21.1)	*NA*
4A	Arr −1,−4,+8,−13,−15,−16,−19,−21,UPD(3)(p21.31-p21.1),UPD(17)(p12-pter)	*NA*
4B	Arr –Y,−2,−7,−8,−11,−13,−22,UPD(1)(p36.13-pter)(q21.3–q42.2),UPD(3)(p26.6-p13)(q24),UPD(4)(p15.31–q21.3),UPD(6)(pter-q23.3),UPD(9)(q21.2–q34.11),UPD(14)(q21.2–q21.3)(q22.3–q32.2),UPD(15)(q15–q15.3),UPD(16)(p12.2–pter)(q22.1–q22.2)(q23.3–q24.1),UPD(17)	*TP73, AIM1, LIN28B, GLIS3, DLK1, MEG3, ZNF597, NAA60, RTL1*
5	Arr−7,−12,−13,UPD(21)	*NA*
6	Arr UPD(1)(p32.2-p32.1),UPD(3)(pter-p26.2)(p24.3-p13)(q12.3-qter)	*NA*
7	Arr UPD(20)(q11.22)	*BLCAP, NNAT*
8	Arr UPD(1–22,X)	*ALL*
